# The Aerobic and Anaerobic Contribution During Repeated 30-s Sprints in Elite Cyclists

**DOI:** 10.3389/fphys.2021.692622

**Published:** 2021-05-26

**Authors:** Nicki Winfield Almquist, Øyvind Sandbakk, Bent R. Rønnestad, Dionne Noordhof

**Affiliations:** ^1^Section for Health and Exercise Physiology, Inland Norway University of Applied Sciences, Lillehammer, Norway; ^2^Centre for Elite Sports Research, Department of Neuromedicine and Movement Science, Norwegian University of Science and Technology, Trondheim, Norway

**Keywords:** GE-method, sprint training, energetic contribution, aerobic and anaerobic power, intermittent exercise

## Abstract

Although the ability to sprint repeatedly is crucial in road cycling races, the changes in aerobic and anaerobic power when sprinting during prolonged cycling has not been investigated in competitive elite cyclists. Here, we used the gross efficiency (GE)-method to investigate: (1) the absolute and relative aerobic and anaerobic contributions during 3 × 30-s sprints included each hour during a 3-h low-intensity training (LIT)-session by 12 cyclists, and (2) how the energetic contribution during 4 × 30-s sprints is affected by a 14-d high-volume training camp with (SPR, *n* = 9) or without (CON, *n* = 9) inclusion of sprints in LIT-sessions. The aerobic power was calculated based on GE determined before, after sprints, or the average of the two, while the anaerobic power was calculated by subtracting the aerobic power from the total power output. When repeating 30-s sprints, the mean power output decreased with each sprint (*p* < 0.001, ES:0.6–1.1), with the majority being attributed to a decrease in mean anaerobic power (first vs. second sprint: −36 ± 15 W, *p* < 0.001, ES:0.7, first vs. third sprint: −58 ± 16 W, *p* < 0.001, ES:1.0). Aerobic power only decreased during the third sprint (first vs. third sprint: −17 ± 5 W, *p* < 0.001, ES:0.7, second vs. third sprint: 16 ± 5 W, *p* < 0.001, ES:0.8). Mean power output was largely maintained between sets (first set: 786 ± 30 W vs. second set: 783 ± 30 W, *p* = 0.917, ES:0.1, vs. third set: 771 ± 30 W, *p* = 0.070, ES:0.3). After a 14-d high-volume training camp, mean power output during the 4 × 30-s sprints increased on average 25 ± 14 W in SPR (*p* < 0.001, ES:0.2), which was 29 ± 20 W more than CON (*p* = 0.008, ES: 0.3). In SPR, mean anaerobic power and mean aerobic power increased by 15 ± 13 W (*p* = 0.026, ES:0.2) and by 9 ± 6 W (*p* = 0.004, ES:0.2), respectively, while both were unaltered in CON. In conclusion, moderate decreases in power within sets of repeated 30-s sprints are primarily due to a decrease in anaerobic power and to a lesser extent in aerobic power. However, the repeated sprint-ability (multiple sets) and corresponding energetic contribution are maintained during prolonged cycling in elite cyclists. Including a small number of sprints in LIT-sessions during a 14-d training camp improves sprint-ability mainly through improved anaerobic power.

## Introduction

Road cycling competitions consist of prolonged low- to moderate-intensity cycling with inclusion of several high-intensity efforts in the decisive moments (Abbiss et al., 2013; van Erp and Sanders, 2020). The ability to perform repeated short-duration (5–15 s), high-intensity efforts is crucial in order to establish a break-away, close a gap or to sprint away from the group and win the race (Abbiss et al., 2013). Recent data from a world-class sprinter has given valuable insight into sprint-finishes, showing a high demand (>500 W) over the last 90 s leading up to a sprint and ~650–900 W over the final 30 s (van Erp et al., 2021a). Accordingly, elite cyclists need to develop both a high aerobic capacity and the ability to repeatedly use anaerobic energy reserves. Indeed, successful, professional cyclists seem able to maintain sprint-ability after prolonged cycling, and to a greater extent than non-successful cyclists (van Erp et al., 2021b). However, the changes in aerobic and anaerobic contributions during repeated sprints and the evolvement hereof during prolonged cycling is yet to be determined in elite cyclists. Such information would improve our understanding of the energetic demands of road cycling competitions where the ability to repeatedly sprint is required.

The aerobic energy contribution can easily be determined by collecting gas-exchange data; however, determining the anaerobic energy contribution relies on indirect methods that are difficult to validate for whole-body exercise. Previously, invasive measures quantifying adenosine triphosphate (ATP), inosine monophosphate (IMP), creatine phosphate (PCr), intermediates of glycolysis, and lactate in muscle biopsies (Bangsbo et al., 1990; Bogdanis et al., 1996) have been applied to determine anaerobic energy contribution in non-elite subjects. However, due to the transient, changeable nature of the anaerobic metabolism and the relatively small muscle samples, this method might not represent the entire active muscle mass. Furthermore, the transferability of previous invasive findings to elite athletes might be questionable and, in addition, the method may be regarded impractical to apply in elite athletes.

The most common indirect methods to determine the anaerobic energy contribution are the maximal accumulated oxygen deficit method (Medbo et al., 1988), the critical power concept (Monod and Scherrer, 1965), and the gross efficiency (GE)-method (Serresse et al., 1988). It is well-established that GE diminishes during prolonged exercise (Hopker et al., 2017; Almquist et al., 2019) in an intensity-dependent manner (Noordhof et al., 2015), affecting performance at the end of a race (Passfield and Doust, 2000; Noordhof et al., 2020). As the GE-method is the only method that takes the decrease in efficiency during exercise into account, this method seems most relevant for determining aerobic/anaerobic contributions during repeated maximal efforts performed during prolonged exercise.

Recently, it has been shown that the repeated sprint-ability of elite cyclists can be improved by including sprints during low-intensity training (LIT)-sessions (Almquist et al., 2020, 2021). However, whether these improvements come from increased aerobic, and/or anaerobic contributions remains elusive. Therefore, the aims of the present study were to: (1) investigate the absolute and relative aerobic and anaerobic contributions of repeated 30-s sprints included during a 3-h low-intensity cycling session using the GE-method, and (2) investigate how the energetic contribution during repeated sprints is affected by a 14-d high-volume training camp where sprints are regularly included in LIT-sessions of elite cyclists. Based on the previous literature, we hypothesized that a decrease in anaerobic power would be the major contributor to the decrease in sprint power when repeating sprints during low-intensity cycling. However, the decrease in sprint power when repeating sets of sprints during prolonged cycling was suggested to be relatively small. Lastly, we hypothesized that the inclusion of 30-s sprints during a 14-d training camp would mainly improve sprint power output through an increase in anaerobic power.

## Methods

### Participants and Experimental Design

The present study included data from two separate studies on elite cyclists of which physiological, performance, body composition, hematological, and muscular measures have been presented previously (Almquist et al., 2019, 2021). Before inclusion in the two separate studies, the participants were made fully aware of the possible risks and discomforts associated with participation. All gave their written informed consent to participate before entering the studies, which were approved by the local ethics committee at Inland Norway University of Applied Sciences and were conducted in accordance with the Declaration of Helsinki.

**Figure 1:**
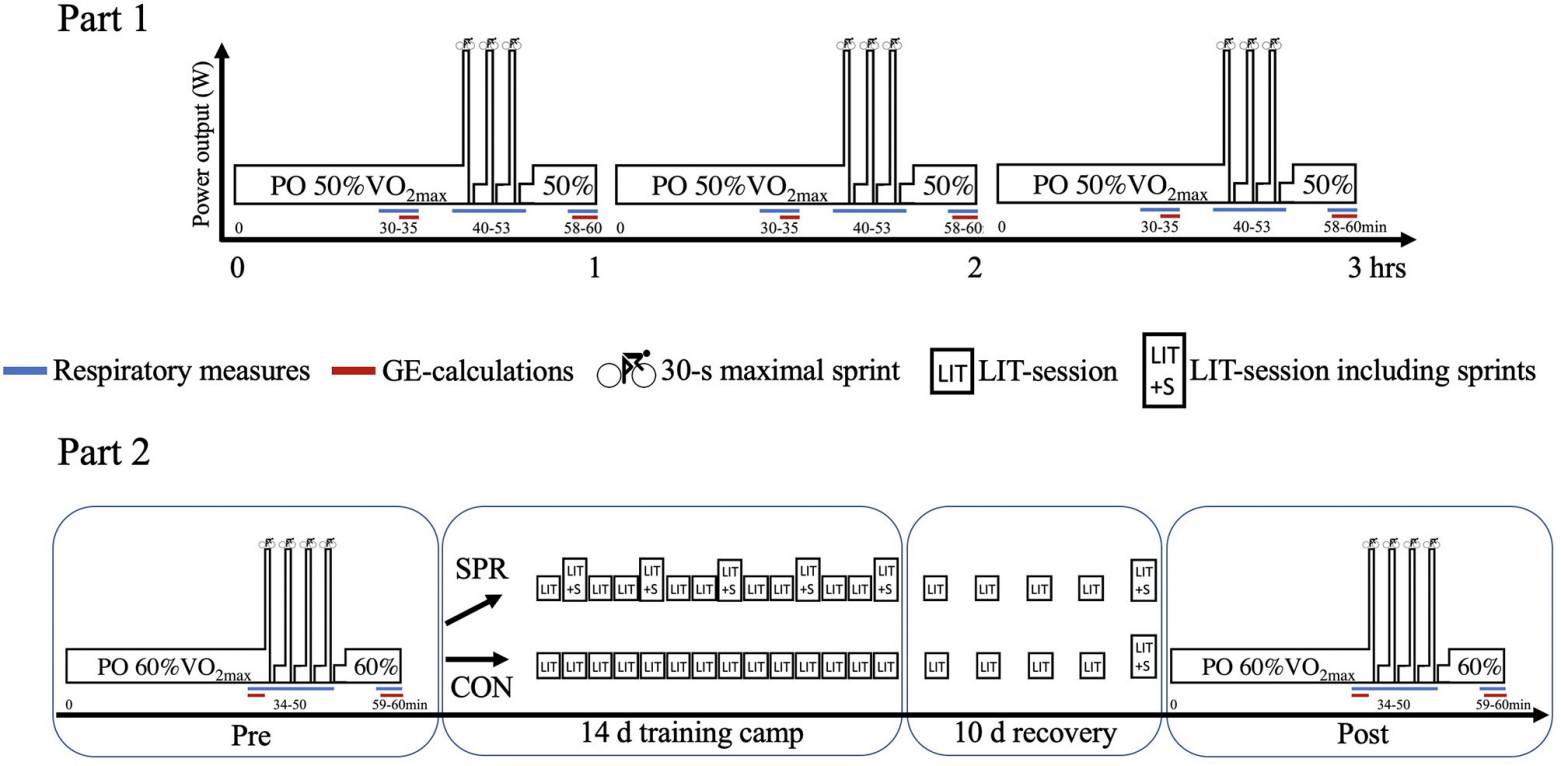
Schematic presentation of Part 1 (upper panel) and Part 2 (lower panel). Part 1 included 12 elite cyclists who performed a 3-h low-intensity training (LIT)-session including three sets of 3 × 30-s maximal sprints interspersed by 4 min recovery. Part 2 included 18 elite cyclists who completed a performance test including 4 × 30-s maximal sprints interspersed by 4 min recovery, which was performed before (Pre) and after (Post) a 14-d training camp and subsequent 10-d recovery period. A control group (CON, *n* = 9) performed only LIT-sessions during the training camp, while a sprint group (SPR, *n* = 9) included 12 × 30-s sprints on five of the LIT-sessions. Both SPR and CON performed a self-administered habituation-exercise trial of ~1 h including 4 × 30-s sprints the day before Pre and Post.

The present study included respiratory and power output data collected during repeated 30-s sprints. By using the GE-method (Noordhof et al., 2013), we were able to calculate novel and previously unpublished data on the energetic contribution during repeated 30-s sprints in elite cyclists. Part 1 of the present study presents the calculated aerobic and anaerobic mean power output and their relative contributions during sets of 3 × 30-s sprints interspersed by 4 min recovery performed each hour during a 3-h LIT-session (Figure 1, Part 1). In Part 2, we present the changes in calculated aerobic and anaerobic mean power output and their relative contributions during 4 × 30-s maximal sprints interspersed by 4 min recovery performed before and after a 14-d LIT-camp. The LIT-camp included 12 × 30-s maximal sprints during five LIT-sessions for the sprint (SPR) group, who were compared to a control group that performed distance-matched LIT-sessions without sprints (CON) (Figure 1, Part 2). The four sets of 3 × 30-s sprints were performed during LIT-sessions with 30–60 min of low-intensity cycling in between sets. Schematic representations including specific time-points of sprints, $\overset{∙}{\text{V}}{\text{O}}_{2}$-measurements, and GE-calculations are shown in Figure 1 and participants' characteristics are presented in Table 1.

**Table 1:** Participants' characteristics and physiological variables determined during an incremental blood lactate profile test and incremental test to exhaustion to determine maximal oxygen consumption ($\overset{∙}{\text{V}}{\text{O}}_{2\text{max}}$).

	**Part 1**	**Part 2**	
	***n* = 12**	**SPR, *n* = 9**	**CON, *n* = 9**
Age (Years)	26.2, 6.3	20.9, 1.4	21.0, 1.7
Body mass (kg)	76.1, 3.2	73.6, 8.4	74.3, 5.0
Height (cm)	183, 5	185, 5	183, 6
Training volume (h·30 d^−1^)	55, 35	55, 16	56, 5
Power output at 4 mmol·L^−1^ [BLa^−1^] (W·kg^−1^)	4.3, 0.6	4.5, 0.3	4.4, 0.4
$\overset{∙}{\text{V}}{\text{O}}_{2\text{max}}$ (mL·kg^−1^·min^−1^)	73.4, 4.0	75.4, 5.2	75.1, 5.4
W_max_ (W·kg^−1^)	6.3, 0.3	6.5, 0.4	6.4, 0.3

*Values are mean ± standard deviation (SD)*.

*$\overset{∙}{\text{V}}{\text{O}}_{2\text{max}}$, Maximal oxygen consumption; W_max_, Maximal power produced during the last minute of an incremental test to exhaustion; SPR, Sprint-group; CON, Control-group*.

### Testing Procedures

The participants reported to the lab for physiological testing on the same time of the day (± 1 h) after at least 2 h of fasting. The participants were instructed to refrain from caffeine, beta-alanine and bicarbonate ingestion 24 h prior to testing and registered and replicated food intake and time of consumption for the last 24 h leading up to the physiological tests. All testing was performed under controlled environmental conditions (16–18°C and 20–35% relative humidity) with a fan ensuring air circulation around the participant. In Part 1, the participants visited the laboratory on two occasions, 1: to perform a blood lactate profile test and a $\overset{∙}{\text{V}}{\text{O}}_{2\text{max}}$ test, and 2: to perform an experimental protocol consisting of 3 h prolonged cycling including 3 sets of 3 × 30-s sprints interspersed by 1 min of passive recovery and 3 min of active recovery. In Part 2, the participants visited the laboratory once and performed in one cohesive 2-hr experimental protocol a blood lactate profile test, a $\overset{∙}{\text{V}}{\text{O}}_{2\text{max}}$ test, and a 60-min prolonged cycling bout including 4 × 30-s sprints with 4 min recovery (1 min passive, 3 min active) in between sprints, while the three distinct tests were interspersed by 10 min of active recovery.

#### Blood Lactate Profile Test and $\overset{∙}{\text{V}}{\text{O}}_{2\text{max}}$ Test

The participants performed a blood lactate profile test followed by an incremental test to exhaustion as described elsewhere (Almquist et al., 2020). Briefly, the participants cycled for 5 min at 175 W, followed by 50-W increments every 5 min until a blood lactate concentration ([BLa^−^]) of 3 mmol·L^−1^, after which increments were 25 W. The test was terminated at a [BLa^−^] of 4 mmol·L^−1^ or higher. After each 5-min increment, capillary blood was sampled from the fingertip and [BLa^−^] was analyzed using a Biosen C line (EKF Diagnostic, Germany). Based on these measures, the power output at 4 mmol·L^−1^ [BLa^−^] was calculated using linear interpolation. After 10-min of active recovery, an incremental test to exhaustion was initiated to determine $\overset{∙}{\text{V}}{\text{O}}_{2\text{max}}$ with 1-min increments, starting at 200–250 W depending on the participants previous results. Power output increased by 25 W·min^−1^ until the participants were unable to maintain a pedaling frequency of >60 revolutions per minute (RPM) despite verbal encouragement from the test leader. Cycling exercise was performed in the seated position on an electromagnetic braked cycle ergometer (Lode Excalibur Sport, Lode B. V., Groningen, The Netherlands). Respiratory data were measured using a computerized metabolic system with a mixing chamber (Oxycon Pro, Erich Jaeger, Hoechberg, Germany) which was calibrated every hour.

#### Experimental Protocols

Part 1: On the second visit, the cyclists rode for 3 h at a power output equivalent to 50% $\overset{∙}{\text{V}}{\text{O}}_{2\text{max}}$, including 3 x 30-s maximal sprints, interspersed by 1 min passive recovery and 3 min active recovery at 100 W (Figure 1). The power output corresponding to 50% $\overset{∙}{\text{V}}{\text{O}}_{2\text{max}}$ was calculated from the blood lactate profile and $\overset{∙}{\text{V}}{\text{O}}_{2\text{max}}$ tests using linear interpolation. Maximal sprints were performed in the seated position using the *Wingate* modus with a flying start from 80 RPM and a resistance of 0.8 Nm·kg^−1^ body mass. During the experimental trial, cyclists consumed water, energy drinks and gels without caffeine (Squeezy Sports Nutrition GmbH, Germany) *ad libitum* to prevent dehydration and glycogen depletion.

Part 2: Ten min after the incremental test, a 60-min continuous cycling test including 4 × 30-s maximal sprints, separated by 1 min passive recovery and 3 min active recovery (100 W), was performed using a similar design as previously described (Almquist et al., 2021) (Figure 1). Due to the relatively short exercise protocol of 60 min in Part 2 compared to Part 1, the participants rode at a power output equivalent to 60% $\overset{∙}{\text{V}}{\text{O}}_{2\text{max}}$ instead of 50% $\overset{∙}{\text{V}}{\text{O}}_{2\text{max}}$. Nutritional intake during the experimental trial before the training camp was similar to Part 1 and was recorded and replicated during the experimental trial after the training camp and subsequent recovery period.

### Data Analysis

$\overset{∙}{\text{V}}{\text{O}}_{2\text{max}}$ was calculated as the highest average of a 1-min moving average using 5-s $\overset{∙}{\text{V}}{\text{O}}_{2}$-measurements. W_max_ was calculated as the mean power output during the last minute of the incremental test. GE was calculated using a two-min average $\overset{∙}{\text{V}}{\text{O}}_{2}$-measurement and respiratory exchange ratio (RER) during a steady-state period riding at a power output of ~50% $\overset{∙}{\text{V}}{\text{O}}_{2\text{max}}$ immediately prior to each set of 3 × 30-s sprints. Likewise for Part 2, GE was calculated using a mean of two-min $\overset{∙}{\text{V}}{\text{O}}_{2}$ measurements and RER during a steady-state period riding at a power output of ~60% of $\overset{∙}{\text{V}}{\text{O}}_{2\text{max}}$ immediately prior to the set of 4 × 30-s sprints (see Figure 1). GE was calculated by dividing power output by power input. Power input was calculated using the oxygen equivalent (Peronnet and Massicotte, 1991), according to equation 1.

(1) $$\begin{array}{r} \text{Power input}={\text{L}\cdot \text{s}}^{-1}\cdot \left(4840\;{\text{J}\cdot \text{L}}^{-1}\cdot \text{RER}+16,890\;{\text{J}\cdot \text{L}}^{-1}\right) \end{array}$$

Due to the circulatory transit delay from the muscles to the lungs when trained cyclists started exercising moderately (Barstow and Mole, 1991), a time-delay of 15 s was applied to the respiratory data, based on previous measures in well-trained cyclists (Mulder et al., 2015). The aerobically attributable mechanical power was determined from the metabolic power input, based on the average respiratory data during the 30-s sprint, and GE. The aerobic contribution during the first 30-s sprint was calculated using the GE determined immediately before the first sprint. The aerobic contribution during the last 30-s sprint was calculated using the GE determined 6 min after the last sprint. Assuming a linear decrease in GE during short sprints (Noordhof et al., 2015), the aerobic contribution during the second sprint was calculated using the average GE from before and after the sprint set. Subsequently, the anaerobically attributable mechanical power was calculated by subtracting the aerobically attributable mechanical power from the mean power output of each 30-s sprint (Serresse et al., 1988; Noordhof et al., 2013).

### Statistical Analyses

To compare absolute (W) and relative (%) changes in the aerobic and anaerobic contribution during repeated sprints and subsequent [BLa^−^], a mixed linear model was applied. For Part 1, to compare main effects of sprint number within each set (1–3) and sprint sets (1–3), a mixed linear model was applied with fixed effects defined by number of sprints, and sprint set and random effects were defined by participant. For Part 2, to compare main effects of group and time a mixed linear model was applied with fixed effects defined by group and time, and random effects were defined by participant. To compare changes from Pre to Post between groups, the absolute and relative changes were corrected using Pre-values as a covariate. Whenever a significant main effect was obtained a Sidak *post hoc* analysis was performed with an alpha-level of 0.05. All statistical analyses were done using SPSS v.25 (IBM Corp, Armonk, NY, USA). Hopkins' ES using pooled SD were calculated to highlight the practical significance of differences in changes between sprints and sets (Part 1) and groups (Part 2). The magnitude of the ES was interpreted as follows: < 0.2 trivial, 0.2–0.6 small, 0.6–1.2 moderate, 1.2–2.0 large, and 2.0–4.0 very large difference (Hopkins et al., 2009).

## Results

### Part 1

#### The Anaerobic and Aerobic Power and Contribution During Sets of 3 × 30-s Sprints

**Figure 2:**
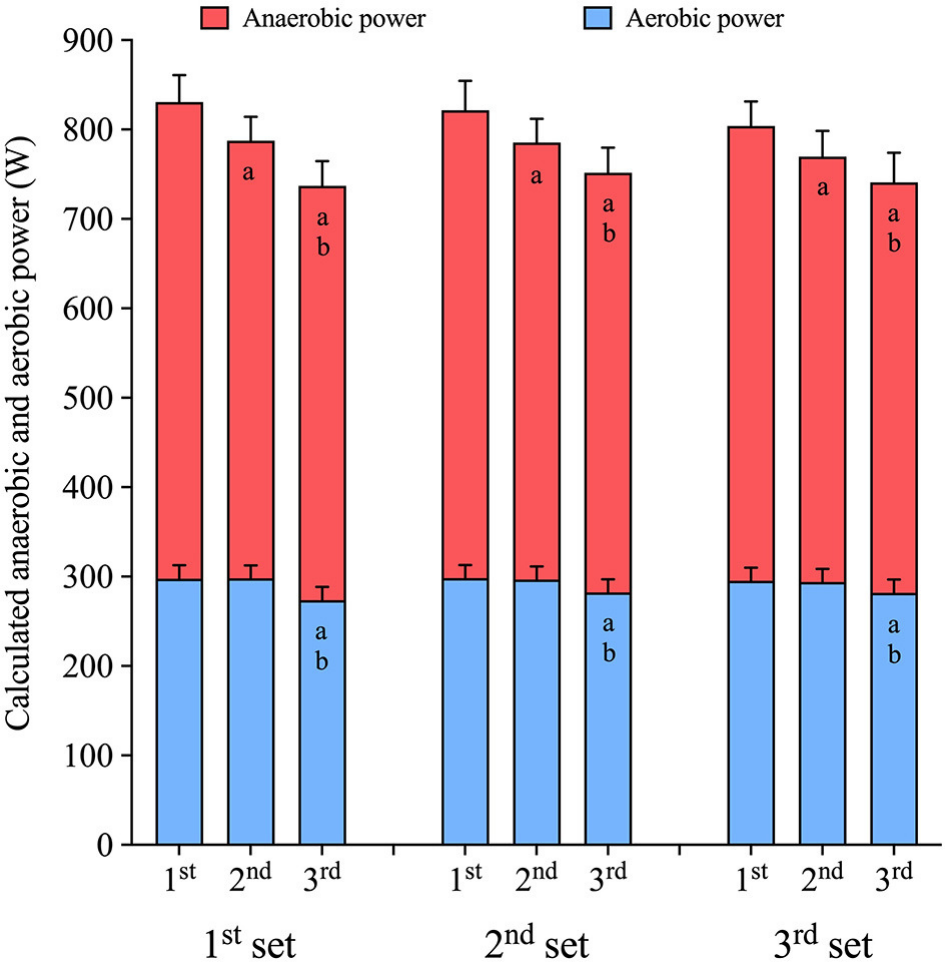
Anaerobic and aerobic mean power output during repeated sets of 3 × 30-s maximal sprints using the GE-method. Sprints were interspersed by 4 min recovery and performed during a 3-h prolonged cycling session riding at a power output equivalent to 50% $\overset{∙}{\text{V}}{\text{O}}_{2\text{max}}$. Data are mean ± 95%CI, *n* = 12. a indicates significant difference from the first sprint (*p* < 0.05). b indicates significant difference from the second sprint (*p* < 0.05).

When repeating 30-s sprints, interspersed by 4 min recovery, the mean power output decreases with each subsequent sprint (*p* < 0.001). A higher mean power output was reached during the first (815 ± 31 W) compared to the second (780 ± 30 W, *p* < 0.001, ES: 0.6) and the third sprint (744 ± 30, *p* < 0.001, ES: 1.1). The decreased mean power was mainly a result of a decrease in mean anaerobic power, which decreased with repeated sprints (*p* < 0.001, Figure 2). The anaerobic power was 36 ± 15 W lower during the second sprint compared to the first sprint (*p* < 0.001, ES: 0.7) and 58 ± 16 W lower during the third sprint compared to the first (*p* < 0.001, ES: 1.0), with the mean anaerobic power also being lower during the third sprint compared to the second sprint (−22 ± 13 W, *p* = 0.001, ES: 0.5). In addition, the aerobic power decreased during the third sprint (*p* < 0.001), being 17 ± 5 W, and 16 ± 5 W lower than during the first (*p* < 0.001, ES: 0.7) and second sprint (*p* < 0.001, ES: 0.8), respectively, without a difference between the first and second sprint (Figure 2).

The relative anaerobic contribution decreased from the first (63.6 ± 1.5%) to the second sprint (62.0 ± 1.5%, *p* < 0.001, ES: 0.6), and from the first to the third sprint (62.4 ± 1.5%, *p* = 0.001, ES: 0.4), while the relative aerobic contribution increased from the first (36.4 ± 1.5%) to the second sprint (38.0 ± 1.5%, *p* < 0.001, ES: 0.6), and from the first to the third sprint (37.6 ± 1.5%, *p* = 0.001, ES: 0.4).

#### The Anaerobic and Aerobic Power and Contribution When Repeating Sets of 30-s Sprints

There was no difference in mean power output between sprint sets (*p* = 0.084). Mean power output was hence maintained from the first set (786 ± 30 W), to the second set (783 ± 30 W, *p* = 0.917, ES: 0.1) and third set (771 ± 30 W, *p* = 0.070, ES: 0.3). Neither mean anaerobic power (first vs. second set: −14 ± 15 W, *p* = 0.060, ES: 0.3, first vs. third set −14 ± 15 W, *p* = 0.058, ES: 0.3), nor mean aerobic power (*p* = 0.485, ES: 0.0) changed between sets of sprints.

The relative anaerobic contribution was unaltered between sets (first set: 63.0 ± 1.5%, second set: 62.7 ± 1.5%, third set: 62.2 ± 1.5%, *p* = 0.083, ES: 0.3), and likewise the aerobic contribution (first set: 37.0 ± 1.5%, second set: 37.3 ± 1.5%, third set: 37.7 ± 1.5%, *p* = 0.083, ES: 0.3).

### Part 2

#### Average Anaerobic and Aerobic Power and Contribution During a Set of Four 30-s Sprints

**Figure 3:**
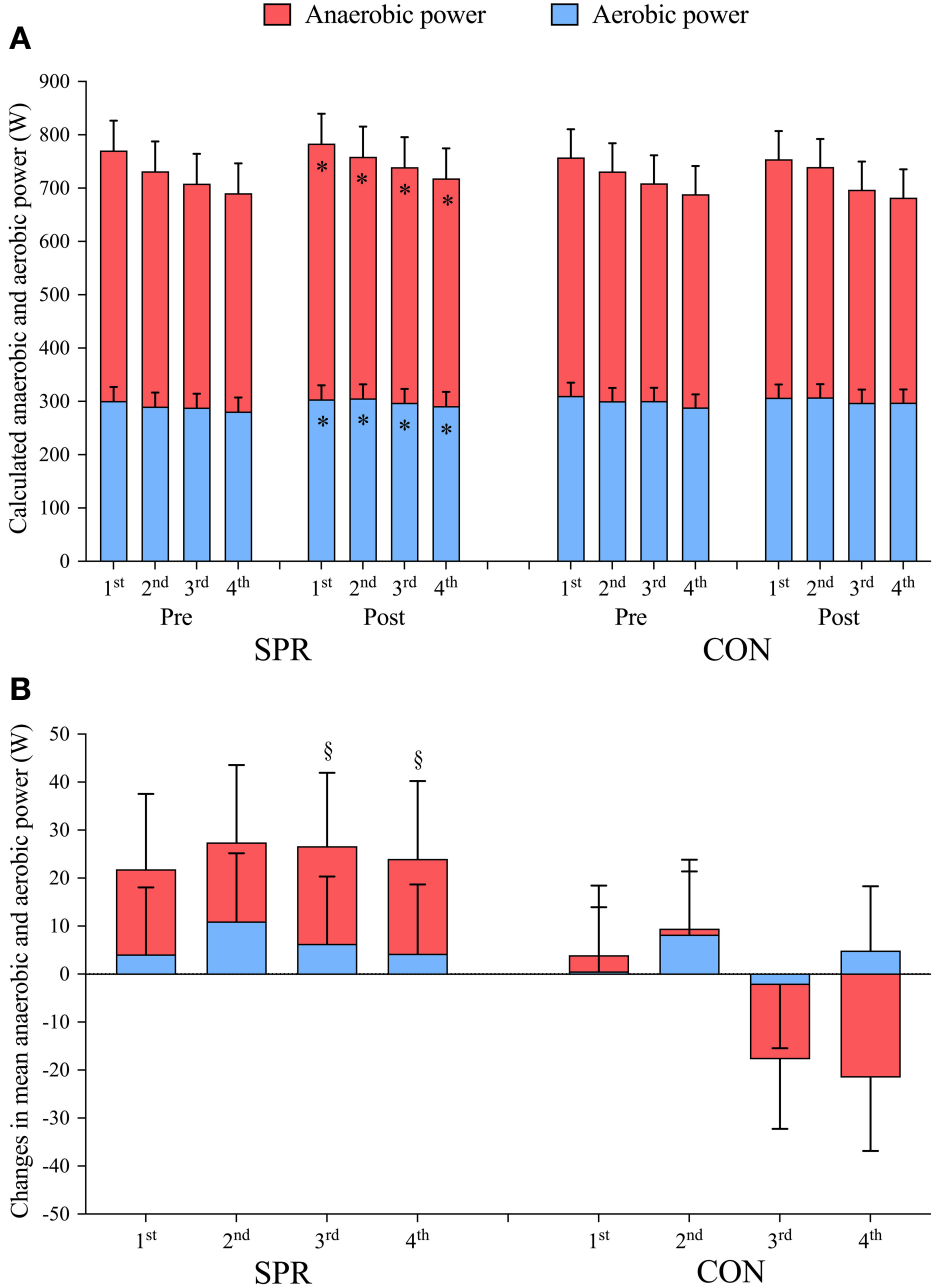
**(A)** Calculated mean anaerobic and aerobic power output during 4 × 30-s maximal sprints interspersed by 4 min recovery performed before (Pre) and after (Post) a 14-d training camp and a 10-d recovery period. **(B)** Changes in mean anaerobic and aerobic power output from Pre to Post. The training camp included daily low-intensity training (LIT) while 12 × 30-s sprints were included during five LIT-sessions (SPR, *n* = 8), compared to only performing LIT (CON, *n* = 9). Data are mean ± 95% CI. * indicates a significant main effect of time (*p* < 0.05). § indicates significant *post-hoc* effects of group on baseline-corrected changes (*p* < 0.05).

As previously presented (Almquist et al., 2021), mean power output during the 4 × 30-s sprints increased on average by 25 ± 14 W in SPR (*p* < 0.001, ES: 0.2) from before to after the training camp, which was 29 ± 20 W more than in CON (*p* = 0.008, ES: 0.3), who maintained sprint power (−4 ± 13 W, *p* = 0.560, ES: 0.1). This improvement in mean sprint power in SPR was due to a 15 ± 13 W increase in mean anaerobic power (*p* = 0.026, ES: 0.2), and a 9 ± 6 W (*p* = 0.004, ES: 0.2) increase in mean aerobic power (Figure 3). Neither anaerobic power (*p* = 0.361, ES: 0.1) nor aerobic power (*p* = 0.413, ES: 0.1) changed in CON from Pre to Post. The increase in mean anaerobic power in SPR was greater than in CON (27 ± 38 W, *p* = 0.004, ES: 0.4). Specifically, the anaerobic power during the third and fourth sprint increased more in SPR from Pre to Post than in CON (3rd: *p* = 0.001, ES: 0.4, 4th: *p* < 0.001, ES: 0.7). Mean aerobic power did not change differently between SPR and CON (4 ± 18 W, *p* = 0.676, ES: 0.1).

There were no differences in the relative anaerobic or aerobic contributions between groups when correcting for baseline values. The relative anaerobic contribution did not change in SPR (Pre: 59.5 ± 3.4% vs. Post: 59.7 ± 3.4%, *p* = 0.727, ES: 0.0) nor CON (Pre: 58.2 ± 3.2% vs. Post: 57.8 ± 3.2%, *p* = 0.315, ES: 0.1) from before to after the training camp. Likewise, the relative aerobic contribution did not change from before to after the training camp in either SPR (Pre: 40.4 ± 3.4% vs. Post: 40.3 ± 3.4%, *p* = 0.727, ES: 0.0) nor CON (Pre: 41.8 ± 3.2% vs. Post: 42.2 ± 3.2%, *p* = 0.315, ES: 0.1).

## Discussion

The aims of the present study were two-fold: (1) to investigate the absolute and relative aerobic and anaerobic contributions of repeated 30-s sprints when included during a 3-h LIT-session using the GE-method, and (2) to investigate how the energetic contribution during repeated sprints is affected by a 14-d high-volume training camp when sprints are regularly included in LIT-sessions. Firstly, we found that when elite cyclists performed three repeated sprints, mean power output decreased with each subsequent sprint. This decrement was, as hypothesized, primarily due to a moderate decrease in anaerobic power with each sprint (ES: 0.7–1.0), but also due to a moderate decrease in aerobic power during the third sprint (ES: 0.7–0.8). Secondly, when performing sets of sprints during a 3-h LIT-session, the mean power output and the corresponding energetic contributions during sprints were largely maintained, which supported our hypothesis. Thirdly, when including sprints during five LIT-sessions on a 14-d training camp, SPR improved 4 × 30-s mean power output by 29 ± 20 W more than CON, who only performed LIT-sessions. In support of our hypothesis, this improvement could mainly be explained by an increase in anaerobic power, particularly during the third and the fourth sprint (ES: 0.4–0.7), compared to CON.

Power output decreases during repeated 30-s maximal sprints with 4 min of recovery in between. The major part of this comes from a decrease in anaerobic power, which was reduced by ~36 W (~7%) from the first to the second sprint, and by ~58 W (~11%) from the first to the third sprint (moderate effects). These moderate decreases are much less than in untrained men, where the anaerobic power during a second 30-s maximal sprint was decreased by 41% when sprints were interspersed by 4 min of passive recovery (Bogdanis et al., 1996). During a 30-s maximal sprint, PCr stores become almost completely depleted (Walter et al., 1997), with the recovery of PCr between sprints being mediated by aerobic ATP-resynthesis, which is following a biexponential pattern (Walter et al., 1997). During passive recovery in untrained subjects, ~80% of PCr rebuilding is accomplished within ~90 s, whereas complete recovery is not obtained before ~10 min (Walter et al., 1997). The recovery kinetics of PCr seem determined by oxygen availability in the recovery period (Haseler et al., 1999). Likewise, recovery of exercise tolerance after maximal efforts is affected by the relative intensity of exercise during an active recovery period (Chidnok et al., 2012). Therefore, a link between training status and faster recovery of both PCr (Takahashi et al., 1995; Tomlin and Wenger, 2001) and anaerobic work capacity in general (represented by W') (Caen et al., 2021) has previously been suggested. Together, this indicates that the high fitness-level of our elite cyclists potentially explains the smaller decreases in anaerobic power compared to untrained men. Although the major part of the decrease in mean power output during repeated sprints came from a decline in anaerobic power, we also found that the aerobic power decreased from the first to the third sprints by ~17 W (~6%, moderate effect). The reduced aerobic power production during repeated sprints might be related to perturbations of ion homeostasis, which have been suggested to lead to fatigue during intense exercise (Hostrup and Bangsbo, 2017). Our findings of the relative aerobic contributions during 30-s sprints being ~36–38% (Part 1) and ~40–42% (Part 2), are in line with previous reports using the accumulated oxygen deficit method in highly trained runners (Spencer and Gastin, 2001). Likewise, invasive studies in untrained men also found an increasing relative aerobic contribution when 30-s sprints are repeated (Bogdanis et al., 1996; Girard et al., 2011). However, in our elite cyclists, only a small increase in the relative aerobic contribution, from ~36% to ~38%, was observed when repeating 30-s sprints. In untrained subjects, the relative aerobic contribution increased from 34 to 49% between the first and a second 30-s sprint (Bogdanis et al., 1996). Of note, the cyclists in the present study started their first sprints from a higher power output ~181 W (see Supplementary Table 1) compared to the second and third sprint, where they exercised at 100 W. The higher $\overset{∙}{\text{V}}{\text{O}}_{2}$ before the first sprint probably explains the relatively high aerobic contribution during the first sprint while a combination of a fast recovery of anaerobic power, a small decrease in absolute aerobic power, and a general reduction in power output explains the rather small changes in relative contribution when repeating sprints.

When repeating sets of 30-s sprints during 3 h prolonged cycling, the mean power output is maintained during the first 2 h but a small (ES: 0.3), non-significant decrease appears in the last set of sprints in the third hour compared to the first. This decrement is caused by a small, non-significant decrease in anaerobic power (ES: 0.3), ultimately increasing the relative aerobic contribution during sprints. These rather small changes in sprint power during prolonged cycling are supported by a recent study where professional, successful cyclists maintained sprint-ability with accumulating levels of work done, and to a greater extent than non-successful cyclists (van Erp et al., 2021b). Ultimately, our data indicate an almost complete recovery of anaerobic power between repeated sets of maximal 30-s sprints, which contributes to maintenance of sprint-ability during prolonged cycling.

Inclusion of a small number of sprints (12 × 30-s during 5 LIT-sessions) during a 14-d LIT-camp improved the average sprint power in SPR compared to CON, which was mainly due to an increased anaerobic power, specifically improving the last two out of four sprints. Our findings (~4% improvement in 2 weeks) are in line with a previous study on recreational runners who increased oxygen debt after a single 30-s sprint by 18% after 8 weeks of sprint training, indicating increased anaerobic work (Nevill et al., 1989). Interestingly, a small increase in mean aerobic power was observed within SPR from before to after the training camp, although this increase was not different from CON. This was somewhat surprising, since 7 weeks of intensified 30-s sprint training in trained cyclists rendered oxygen kinetics unchanged (Christensen et al., 2015). However, this discrepancy might be explained by the greater increase in $\overset{∙}{\text{V}}{\text{O}}_{2}$ measured 30-s prior to sprinting in SPR compared to CON after the training camp (see details in our Supplementary Table 2). An elevated $\overset{∙}{\text{V}}{\text{O}}_{2}$ prior to sprinting could also reflect a greater re-loading of oxygen bound to myoglobin (Astrand et al., 1960; Richardson et al., 1995), buffering the initial oxygen demand with repeated sprinting. Furthermore, the improved aerobic power within SPR could reflect adaptations to the high aerobic stress during long 30-s sprints (Buchheit et al., 2012), highlighting the relevance of such long sprints for both anaerobic and aerobic energy system development. Still, the improved sprint performance after a period of sprint training was mainly ascribed to an improved anaerobic power.

### Practical Applications

The present findings show that the decrease in sprint power during repeated sprints stems mainly from a decreased anaerobic power, although, aerobic power was also decreased during the third sprint. This yields valuable insight into the energetics of repeated sprinting in elite cyclists which seems to differ from untrained or physically active subjects, providing important practical insight into fitness-level differences in energetic contributions to intense exercise. Mathematical quantification of the depletion and reconstitution of the anaerobic capacity has previously been investigated in physically active subjects (Skiba et al., 2012; Chidnok et al., 2013). Our findings of altered anaerobic and aerobic power contributions when repeatedly sprinting during prolonged cycling, therefore, ought to be taken into account when modeling anaerobic energy use in the future, something that is done in sports science, but also in sports practice. Of practical interest is also the small decrease in anaerobic power between sprints in elite cyclists, indicating that elite cyclists are able to attack several times during a race without risking a substantial decrease in sprint-ability later on. Inclusion of sprints during habitual LIT-sessions shows rapid improvements in the repeated ability to produce anaerobic power and thereby enhances sprint-ability in elite cyclists (Almquist et al., 2020, 2021; Taylor et al., 2021). This improved ability may be important to optimize for tactical reasons, and coaches should therefore consider tracking cyclists' individual, repeated sprint-ability during the season.

### Limitations

Although the present findings yield valuable information regarding the energetics of repeated sprinting during low-intensity endurance exercise, the study design also includes some limitations. To ensure high quality in our VO_2_ and power output measures, all tests were performed on gold-standard laboratory equipment. However, to increase ecologic validity, these measures would benefit from being performed in real-life sprint-sessions i.e., through application of wearable respiratory apparatus' and on-bike power meters.

## Conclusions

Mean sprint power decreases during repeated 30-s sprints, which primarily relates to a decrease in anaerobic power although aerobic power also decreases slightly. However, the rather fast recovery of anaerobic power leads to only moderate decreases in sprint power during sets and maintains sprint-ability and corresponding energetic contributions when sets of sprints are repeated during prolonged cycling in elite cyclists. Finally, including a small number of sprints during LIT-sessions within a 14-d training camp improves sprint-ability mainly through improved anaerobic power.

## Data Availability Statement

The original contributions presented in the study are included in the article/Supplementary Material, further inquiries can be directed to the corresponding author.

## Ethics Statement

The studies involving human participants were reviewed and approved by Local ethics committee at Inland Norway University of Applied Sciences. The patients/participants provided their written informed consent to participate in this study.

## Author Contributions

NA, ØS, and BR contributed to the conception and design of the study. NA executed the study and collected data. NA and DN performed statistical analyses and wrote the draft of the manuscript. All authors contributed to manuscript revision, read, and approved the submitted version.

## Conflict of Interest

The authors declare that the research was conducted in the absence of any commercial or financial relationships that could be construed as a potential conflict of interest.

## References

[B1] Abbiss C. R. Menaspa P. Villerius V. Martin D. T. (2013). Distribution of power output when establishing a breakaway in cycling. Int. J. Sports Physiol. Perform. 8, 452–455. 10.1123/ijspp.8.4.452 23539668

[B2] Almquist N. W. Ettema G. Hopker J. Sandbakk O. Ronnestad B. R. (2019). The effect of 30-second sprints during prolonged exercise on gross efficiency, electromyography, and pedaling technique in elite cyclists. Int. J. Sports Physiol. Perform. 15, 1–9. 10.1123/ijspp.2019-0367 31693997

[B3] Almquist N. W. Løvlien I. Byrkjedal P. T. Spencer M. Kristoffersen M. Skovereng K.. (2020). Effects of including sprints in one weekly low-intensity training session during the transition period of elite cyclists. Front. Physiol. 11:1000. 10.3389/fphys.2020.01000 33041839 PMC7518025

[B4] Almquist N. W. Wilhelmsen M. Ellefsen S. Sandbakk O. Ronnestad B. R. (2021). Inclusion of 30-s sprints during low-intensity sessions during a training camp improves performance in elite cyclists. Med. Sci. Sports Exerc. 10.1249/MSS.0000000000002709 34081058

[B5] Astrand I. Astrand P. O. Christensen E. H. Hedman R. (1960). Myohemoglobin as an oxygen-store in man. Acta Physiol. Scand. 48, 454–460. 10.1111/j.1748-1716.1960.tb01880.x 13794891

[B6] Bangsbo J. Gollnick P. D. Graham T. E. Juel C. Kiens B. Mizuno M.. (1990). Anaerobic energy production and O2 deficit-debt relationship during exhaustive exercise in humans. J. Physiol. 422, 539–559. 10.1113/jphysiol.1990.sp018000 2352192 PMC1190148

[B7] Barstow T. J. Mole P. A. (1991). Linear and nonlinear characteristics of oxygen uptake kinetics during heavy exercise. J Appl Physiol (1985). 71, 2099–2106. 10.1152/jappl.1991.71.6.2099 1778898

[B8] Bogdanis G. C. Nevill M. E. Boobis L. H. Lakomy H. K. (1996). Contribution of phosphocreatine and aerobic metabolism to energy supply during repeated sprint exercise. J. Appl. Physiol. (1985) 80, 876–884. 10.1152/jappl.1996.80.3.876 8964751

[B9] Buchheit M. Abbiss C. R. Peiffer J. J. Laursen P. B. (2012). Performance and physiological responses during a sprint interval training session: relationships with muscle oxygenation and pulmonary oxygen uptake kinetics. Eur. J. Appl. Physiol. 112, 767–779. 10.1007/s00421-011-2021-1 21667291

[B10] Caen K. Bourgois G. Dauwe C. Blancquaert L. Vermeire K. Lievens E.. (2021). W' recovery kinetics following exhaustion: a two-phase exponential process influenced by aerobic fitness. Med. Sci. Sports Exerc. 10.1249/MSS.0000000000002673. [Epub ahead of print]. 33787532

[B11] Chidnok W. Dimenna F. J. Bailey S. J. Vanhatalo A. Morton R. H. Wilkerson D. P.. (2012). Exercise tolerance in intermittent cycling: application of the critical power concept. Med. Sci. Sports Exerc. 44, 966–976. 10.1249/MSS.0b013e31823ea28a 22033512

[B12] Chidnok W. DiMenna F. J. Fulford J. Bailey S. J. Skiba P. F. Vanhatalo A.. (2013). Muscle metabolic responses during high-intensity intermittent exercise measured by (31)P-MRS: relationship to the critical power concept. Am. J. Physiol. Regul. Integr. Comp. Physiol. 305, R1085–R1092. 10.1152/ajpregu.00406.2013 24068048 PMC3840318

[B13] Christensen P. M. Gunnarsson T. P. Thomassen M. Wilkerson D. P. Nielsen J. J. Bangsbo J. (2015). Unchanged content of oxidative enzymes in fast-twitch muscle fibers and V O2 kinetics after intensified training in trained cyclists. Physiol. Rep. 3:e12428. 10.14814/phy2.12428 26152692 PMC4552518

[B14] Girard O. Mendez-Villanueva A. Bishop D. (2011). Repeated-sprint ability–part I: factors contributing to fatigue. Sports Med. 41, 673–694. 10.2165/11590550-000000000-00000 21780851

[B15] Haseler L. J. Hogan M. C. Richardson R. S. (1999). Skeletal muscle phosphocreatine recovery in exercise-trained humans is dependent on O2 availability. J. Appl. Physiol. (1985) 86, 2013–2018. 10.1152/jappl.1999.86.6.2013 10368368

[B16] Hopker J. G. O'Grady C. Pageaux B. (2017). Prolonged constant load cycling exercise is associated with reduced gross efficiency and increased muscle oxygen uptake. Scand. J. Med. Sci. Sports 27, 408–417. 10.1111/sms.12673 26993076

[B17] Hopkins W. G. Marshall S. W. Batterham A. M. Hanin J. (2009). Progressive statistics for studies in sports medicine and exercise science. Med. Sci. Sports Exerc. 41, 3–13. 10.1249/MSS.0b013e31818cb278 19092709

[B18] Hostrup M. Bangsbo J. (2017). Limitations in intense exercise performance of athletes–effect of speed endurance training on ion handling and fatigue development. J. Physiol. 595, 2897–2913. 10.1113/JP273218 27673449 PMC5407979

[B19] Medbo J. I. Mohn A. C. Tabata I. Bahr R. Vaage O. Sejersted O. M. (1988). Anaerobic capacity determined by maximal accumulated O2 deficit. J. Appl. Physiol. (1985) 64, 50–60. 10.1152/jappl.1988.64.1.50 3356666

[B20] Monod H. Scherrer J. (1965). The work capacity of a synergic muscular group. Ergonomics 8, 329–338. 10.1080/00140136508930810

[B21] Mulder R. C. Noordhof D. A. Malterer K. R. Foster C. de Koning J. J. (2015). Anaerobic work calculated in cycling time trials of different length. Int. J. Sports Physiol. Perform. 10, 153–159. 10.1123/ijspp.2014-0035 24911592

[B22] Nevill M. E. Boobis L. H. Brooks S. Williams C. (1989). Effect of training on muscle metabolism during treadmill sprinting. J. Appl. Physiol. (1985) 67, 2376–2382. 10.1152/jappl.1989.67.6.2376 2606844

[B23] Noordhof D. A. Mulder R. C. Malterer K. R. Foster C. de Koning J. J. (2015). The decline in gross efficiency in relation to cycling time-trial length. Int. J. Sports Physiol. Perform. 10, 64–70. 10.1123/ijspp.2014-0034 24911784

[B24] Noordhof D. A. Ofsteng S. J. Nirenberg L. Hammarstrom D. Hansen J. Ronnestad B. R.. (2020). Performance-determining variables in long-distance events: should they be determined from a rested state or after prolonged submaximal exercise? Int. J. Sports Physiol. Perform. 16, 647–654. 10.1123/ijspp.2019-0987 33291068

[B25] Noordhof D. A. Skiba P. F. de Koning J. J. (2013). Determining anaerobic capacity in sporting activities. Int. J. Sports Physiol. Perform. 8, 475–482. 10.1123/ijspp.8.5.475 24026759

[B26] Passfield L. Doust J. H. (2000). Changes in cycling efficiency and performance after endurance exercise. Med. Sci. Sports Exerc. 32, 1935–1941. 10.1097/00005768-200011000-00018 11079525

[B27] Peronnet F. Massicotte D. (1991). Table of nonprotein respiratory quotient: an update. Can. J. Sport Sci. 16, 23–29. 1645211

[B28] Richardson R. S. Noyszewski E. A. Kendrick K. F. Leigh J. S. Wagner P. D. (1995). Myoglobin O2 desaturation during exercise. evidence of limited O2 transport. J. Clin. Invest. 96, 1916–1926. 10.1172/JCI118237 7560083 PMC185828

[B29] Serresse O. Lortie G. Bouchard C. Boulay M. R. (1988). Estimation of the contribution of the various energy systems during maximal work of short duration. Int. J. Sports Med. 9, 456–460. 10.1055/s-2007-1025051 3253239

[B30] Skiba P. F. Chidnok W. Vanhatalo A. Jones A. M. (2012). Modeling the expenditure and reconstitution of work capacity above critical power. Med. Sci. Sports Exerc. 44, 1526–1532. 10.1249/MSS.0b013e3182517a80 22382171

[B31] Spencer M. R. Gastin P. B. (2001). Energy system contribution during 200- to 1500-m running in highly trained athletes. Med. Sci. Sports Exerc. 33, 157–162. 10.1097/00005768-200101000-00024 11194103

[B32] Takahashi H. Inaki M. Fujimoto K. Katsuta S. Anno I. Niitsu M.. (1995). Control of the rate of phosphocreatine resynthesis after exercise in trained and untrained human quadriceps muscles. Eur. J. Appl. Physiol. Occup. Physiol. 71, 396–404. 10.1007/BF00635872 8565970

[B33] Taylor M. Almquist N. W. Ronnestad B. R. Tjonna A. E. Kristoffersen M. Spencer M.. (2021). The inclusion of sprints in low-intensity sessions during the transition period of elite cyclists improves endurance performance 6 weeks into the subsequent preparatory period. Int. J. Sports Physiol. Perform. 10.1123/ijspp.2020-0594. [Epub ahead of print]. 33819914

[B34] Tomlin D. L. Wenger H. A. (2001). The relationship between aerobic fitness and recovery from high intensity intermittent exercise. Sports Med. 31, 1–11. 10.2165/00007256-200131010-00001 11219498

[B35] van Erp T. Kittel M. Lamberts R. P. (2021a). Sprint tactics in the tour de france: a case study of a world-class sprinter (Part II). Int. J. Sports Physiol. Perform. 10.1123/ijspp.2020-0701. [Epub ahead of print]. 33561820

[B36] van Erp T. Sanders D. (2020). Demands of professional cycling races: influence of race category and result. Eur. J. Sport Sci. 10.1080/17461391.2020.1788651. [Epub ahead of print]. 32584197

[B37] van Erp T. Sanders D. Lamberts R. P. (2021b). Maintaining power output with accumulating levels of work done is a key determinant for success in professional cycling. Med. Sci. Sports Exerc. 10.1249/MSS.0000000000002656. [Epub ahead of print]. 33731651

[B38] Walter G. Vandenborne K. McCully K. K. Leigh J. S. (1997). Noninvasive measurement of phosphocreatine recovery kinetics in single human muscles. Am. J. Physiol. 272(2 Pt 1), C525–C534. 10.1152/ajpcell.1997.272.2.C525 9124295

