# Supplementary material for: The Aerobic and Anaerobic Contribution During Repeated 30-s Sprints in Elite Cyclists
Source: Front Physiol. 2021 May 26;12:692622. doi: 10.3389/fphys.2021.692622 (PMC8187900; doi:10.3389/fphys.2021.692622)
Supplement: Supplementary file 1 [file Data_Sheet_1.docx]

Supplementary material

Table 1: Oxygen consumption ($\dot{V}O_{2})$prior to each sprint and gross efficiency (GE) used for calculating aerobic and anaerobic contributions. $\dot{V}$O_2_ was measured 30-s prior to each sprint. Prior to the first sprint, the participants exercised at ~50%$\dot{V}$O_2max_, while prior to second and third sprint, they exercised at 100 W. The GE used for calculating aerobic and anaerobic contributions were determined 7-9 min prior to the first sprint and 6-7 min after the last sprint and the intermediate GE was calculated as an average of these. a = significant post hoc difference from the first sprint in the set (p<0.005), b = significant post hoc difference from the second sprint in the set (p<0.005).

| Part 1 | 1^st^ set | | | 2^nd^ set | | | 3^rd^ set | | |
| --- | --- | --- | --- | --- | --- | --- | --- | --- | --- |
|  | 1^st^ sprint | 2^nd^ sprint | 3^rd^ sprint | 1^st^ sprint | 2^nd^ sprint | 3^rd^ sprint | 1^st^ sprint | 2^nd^ sprint | 3^rd^ sprint |
| GE (%) | 18.6±0.5 | 17.9±0.5a | 17.2±0.5a,b | 18.5±0.5 | 18.0±0.4a | 17.3±0.5a,b | 18.5±0.5 | 17.9±0.5a | 17.2±0.5a |
| $\dot{V}O_{2}$ (mL·min^-1^) | 3097±170 | 2454±134a | 2465±133a | 3136±162 | 2399±109a | 2482±112a | 3078±152 | 2402±114a | 2385±137a |
| Power output (W) | 181±15 | 100 ± 0a | 100 ± 0a | 181±15 | 100 ± 0a | 100 ± 0a | 181±15 | 100 ± 0a | 100 ± 0a |

GE; Gross efficiency, $\dot{V}O_{2}$; Oxygen consumption, SPR; Sprint-group, CON; Control-group.

Table 2: Oxygen consumption ($\dot{V}O_{2})$ and ventilation ($\dot{\mathrm{VE}})$ prior to each sprint and gross efficiency (GE) used for calculating aerobic and anaerobic contributions. $\dot{V}O_{2}$, $\dot{\mathrm{VE}}$, respiratory exchange ratio (RER), revolutions per minute (RPM), and power were measured 30-s prior to each sprint. Prior to the first sprint, the participants exercised at ~60%$\dot{V}O_{2}$_max_, while prior to sprint 2, 3, and 4 they exercised at 100 W. The GE used for calculating aerobic and anaerobic contributions were determined 2 min prior to the first sprint and 9-10 min after the last sprint. GE for sprint 2 and 3 were calculated as an average of these. * indicates significant post hoc effect of time (p<0.005). § indicates significant post hoc interaction of time x group (p<0.005). a indicates significant post hoc difference from the first sprint (p<0.005).

| Part 2 |  | SPR | | | | |  | CON | | | |
| --- | --- | --- | --- | --- | --- | --- | --- | --- | --- | --- | --- |
|  |  | 1^st^ sprint | 2^nd^ sprint | 3^rd^ sprint | 4^th^ sprint |  | 1^st^ sprint | | 2^nd^ sprint | 3^rd^ sprint | 4^th^ sprint |
| GE (%) | Pre | 19.2±0.6 | 19.1±0.6 | 19.1±0.6 | 19.0±0.6 |  | 19.4±0.6 | | 19.3±0.6 | 19.3±0.6 | 19.2±0.6 |
|  | Post | 18.9±0.6 | 18.6±0.6* | 18.6±0.6* | 18.3±0.6* |  | 18.9±0.6* | | 18.8±0.6* | 18.8±0.6* | 18.7±0.6* |
| $\dot{V}O_{2}$ (mL·min^-1^) | Pre | 3563±215 | 2293±215a | 2294±215a | 2307±215a |  | 3550±202 | | 2385±202a | 2359±202a | 2342±202a |
|  | Post | 3637±215 | 2566±221*§a | 2403±215a | 2387±215a |  | 3517±202 | | 2293±202a | 2327±202a | 2304±207a |
| $\dot{V}E$ (L·min^-1^) | Pre | 95±11 | 85±11 | 90±11 | 98±11 |  | 95±11 | | 90±1 | 95±11 | 101±11 |
|  | Post | 97±11 | 97±11* | 100±11 | 106±11§ |  | 91±11 | | 82±11 | 90±11 | 90±11* |
| RER ($\dot{V}$CO_2_·$\dot{V}$O_2_^-1^) | Pre | 0.96±0.04 | 1.08±0.04 | 1.03±0.04 | 1.02±0.04 |  | 0.94±0.04 | | 1.08±0.04 | 1.04±0.04 | 1.02±0.04 |
|  | Post | 0.96±0.04 | 1.11±0.04 | 1.07±0.04* | 1.07±0.04*§ |  | 0.94±0.04 | | 1.06±0.04 | 1.03±0.04 | 1.00±0.04 |
| RPM | Pre | 83±2 | 82±2 | 83±2 | 82±2 |  | 81±2 | | 81±2 | 81±2 | 81±2 |
|  | Post | 82±2 | 83±2 | 82±2 | 82±2 |  | 81±2 | | 80±2 | 82±2 | 80±2 |
| Power output (W) | Pre | 234±32 | 100±0a | 100±0a | 100±0a |  | 235±23 | | 100±0a | 100±0a | 100±0a |
|  | Post | 234±32 | 100±0a | 100±0a | 100±0a |  | 235±23 | | 100±0a | 100±0a | 100±0a |

GE; Gross efficiency, $\dot{V}O_{2}$; Oxygen consumption, $\dot{V}$E; Ventilation, RER; Respiratory exchange ratio, RPM; Revolutions per minute, SPR; Sprint-group, CON; Control-group.
